# Direct assessment of extracerebral signal contamination on optical measurements of cerebral blood flow, oxygenation, and metabolism

**DOI:** 10.1117/1.NPh.7.4.045002

**Published:** 2020-10-07

**Authors:** Daniel Milej, Androu Abdalmalak, Ajay Rajaram, Keith St. Lawrence

**Affiliations:** a Lawson Health Research Institute, Imaging Program, London, Ontario, Canada; b Western University, Department of Medical Biophysics, London, Ontario, Canada

**Keywords:** time-resolved measurements, near-infrared spectroscopy, dynamic contrast-enhanced measurements, diffuse correlation spectroscopy, diffuse reflectance, indocyanine green, brain imaging

## Abstract

**Significance:** Near-infrared spectroscopy (NIRS) combined with diffuse correlation spectroscopy (DCS) provides a noninvasive approach for monitoring cerebral blood flow (CBF), oxygenation, and oxygen metabolism. However, these methods are vulnerable to signal contamination from the scalp. Our work evaluated methods of reducing the impact of this contamination using time-resolved (TR) NIRS and multidistance (MD) DCS.

**Aim:** The magnitude of scalp contamination was evaluated by measuring the flow, oxygenation, and metabolic responses to a global hemodynamic challenge. Contamination was assessed by collecting data with and without impeding scalp blood flow.

**Approach:** Experiments involved healthy participants. A pneumatic tourniquet was used to cause scalp ischemia, as confirmed by contrast-enhanced NIRS, and a computerized gas system to generate a hypercapnic challenge.

**Results:** Comparing responses acquired with and without the tourniquet demonstrated that the TR-NIRS technique could reduce scalp contributions in hemodynamic signals up to 4 times ($r_{\mathrm{SD}}=3\;\mathrm{cm}$) and 6 times ($r_{\mathrm{SD}}=4\;\mathrm{cm}$). Similarly, blood flow responses from the scalp and brain could be separated by analyzing MD DCS data with a multilayer model. Using these techniques, there was no change in metabolism during hypercapnia, as expected, despite large increases in CBF and oxygenation.

**Conclusion:** NIRS/DCS can accurately monitor CBF and metabolism with the appropriate enhancement to depth sensitivity, highlighting the potential of these techniques for neuromonitoring.

## 1 Introduction

Near-infrared spectroscopy (NIRS) has long been considered ideal for brain monitoring of critical-care patients given its intrinsic sensitivity to tissue oxygenation, portability, safety, and low cost.^1^^,^^2^ Although the overwhelming focus of clinical applications of commercial, continuous-wave (CW) NIRS systems has been on tissue oxygen saturation (${\mathrm{StO}}_{2}$), the increasing interest in diffuse correlation spectroscopy (DCS) has opened the possibility to monitor cerebral blood flow (CBF) in conjunction with ${\mathrm{StO}}_{2}$.^3^ This combination can be used to determine the cerebral metabolic rate of oxygen (${\mathrm{CMRO}}_{2}$), which has been applied to both neonatal and adult critical-care patients.^4^^,^^5^ Furthermore, incorporating dynamic contrast-enhanced (DCE) NIRS, which uses the light-absorbing dye indocyanine green (ICG) as an intravascular contrast agent, enables the blood flow index (BFI) from DCS to be converted into perfusion units,^6^^–^^8^ which can be used to quantify ${\mathrm{CMRO}}_{2}$ as well.^9^^,^^10^

One of the main and well-known challenges with adapting these optical neuromonitoring techniques to adult patients is dealing with limited depth sensitivity, which is caused by substantially greater light interactions in superficial tissues (i.e., scalp and skull) compared with the brain. As a consequence, fluctuations in scalp hemodynamics can overshadow brain-related signals,^11^ and quantification of cerebral hemodynamics and metabolism requires accounting for signal contributions from the extracerebral layer (ECL). Ignoring these contributions can result in substantial errors in ${\mathrm{StO}}_{2}$^12^^,^^13^ and CBF.^14^^–^^16^ To reduce the influence of ECL contamination, most commercial CW-NIRS devices subtract signals measured at two source–detector distances. This approach relies on the assumption that scalp contributions will be similar at the two distances; however, this can be altered by factors such as local variations in scalp hemodynamics and skin-probe contact.^17^ A number of approaches, such as principal component analysis and independent component analysis,^18^ and modeling, such as Monte Carlo simulations,^19^ have been proposed to eliminate ECL artifacts, but a standardized method remains an active research area.

An alternative approach for addressing this issue is to use NIRS techniques that can enhance depth sensitivity, such as frequency-domain or time-resolved (TR) methods.^20^^–^^24^ Of the two, TR-NIRS provides the greatest depth information by collecting the distribution of times-of-flight (DTOF) of diffusely reflected photons. Based on the principle that time is proportional to distance, photons that only interrogate the ECL will be recorded earlier than photons that travel deeper into brain tissue. Consequently, TR detection can substantially improve the sensitivity to the brain by focusing on late-arriving photons.^25^^–^^27^ This approach has been shown to provide superior detection sensitivity in functional NIRS applications^28^^–^^30^ and the ability to quantify CBF when adapted to DCE-NIRS.^31^^,^^32^ Although TR detection has also been proposed for DCS,^33^ neuromonitoring applications of this approach are challenging due to the poorer signal-to-noise ratio (SNR) of current technology.^34^ However, the substantially higher blood flow in the brain compared with the scalp gives DCS an inherent advantage in terms of depth sensitivity compared with CW NIRS.^15^ Studies involving tissue-mimicking phantoms, animal models, and human applications have shown that scalp and CBF can be separated using multilayered analytical models to analyze DCS data collected at different source–detector separations.^16^^,^^35^^–^^37^

These studies suggest that, with the appropriate depth-enhancing techniques, NIRS and DCS can accurately monitor ${\mathrm{StO}}_{2}$ and CBF, respectively, and by extension, ${\mathrm{CMRO}}_{2}$. However, the sensitivities of TR-NIRS and multidistance (MD) DCS to the ECL when applied to the adult head have not been rigorously tested due to the difficulties of manipulating scalp blood flow (SBF) independently from cerebral hemodynamics. To address this point, this study presents tissue oxygenation and BFI data collected in response to hypercapnia. Hypercapnia, which refers to an increase in arterial carbon dioxide tension, can be induced by breathing a gas mixture with an elevated concentration of carbon dioxide (${\mathrm{CO}}_{2}$).^38^ Previous studies have shown that hypercapnia increases CBF by its vasodilatory effect on the cerebral vasculature.^39^^–^^41^ A number of studies have shown that the cerebrovascular effects of hypercapnia can be monitored by NIRS.^42^^,^^43^ To evaluate the confounding effects of changes in scalp hemodynamics, data were acquired with and without temporarily restricting blood flow to the scalp by a pneumatic tourniquet wrapped around the head.^44^ The effects of the tourniquet on SBF were confirmed by DCE-NIRS. To compare tissue oxygenation and BFI responses from the two trials, a computerized gas control system was used to generate a reproducible hypercapnic challenge.^45^ Because this stimulus causes vasodilation throughout the brain, it avoids potential partial volume errors associated with detecting focal activation. In addition, it provides a means of evaluating if the measured changes in ${\mathrm{StO}}_{2}$ and CBF are accurate, considering there should be no corresponding change in ${\mathrm{CMRO}}_{2}$.^46^ To assess depth sensitivity, the TR-NIRS data were acquired at a short source–detector distance ($r_{\mathrm{SD}}$) of 1 cm, which is predominately sensitivity to the scalp, and two longer distances ($r_{\mathrm{SD}}=3$ and 4 cm) to increase the sensitivity to the brain. In addition, moment analysis was applied to recorded DTOFs since higher moments are more sensitive to late-arriving photons due to the right skewness of these distributions.^47^^,^^48^ Similarly, DCS data were recorded at $r_{\mathrm{SD}}$ equal to 1 and 3 cm and analyzed using a multilayer solution to the diffusion approximation to separate scalp and brain blood flow.^16^

## 2 Methods

Five healthy subjects (5 males, aged 25 to 36 years, mean=28±4 years) with no history of any neurological or psychiatric disorders were recruited. Written informed consent was obtained from all participants, and all protocols/procedures were approved by the Western University Health Sciences Research Ethics Board, which adheres to the guidelines of the Tri-Council Policy Statement, Ethical Conduct for Research Involving Humans.

### 2.1 Experimental Design

**Fig. 1:**
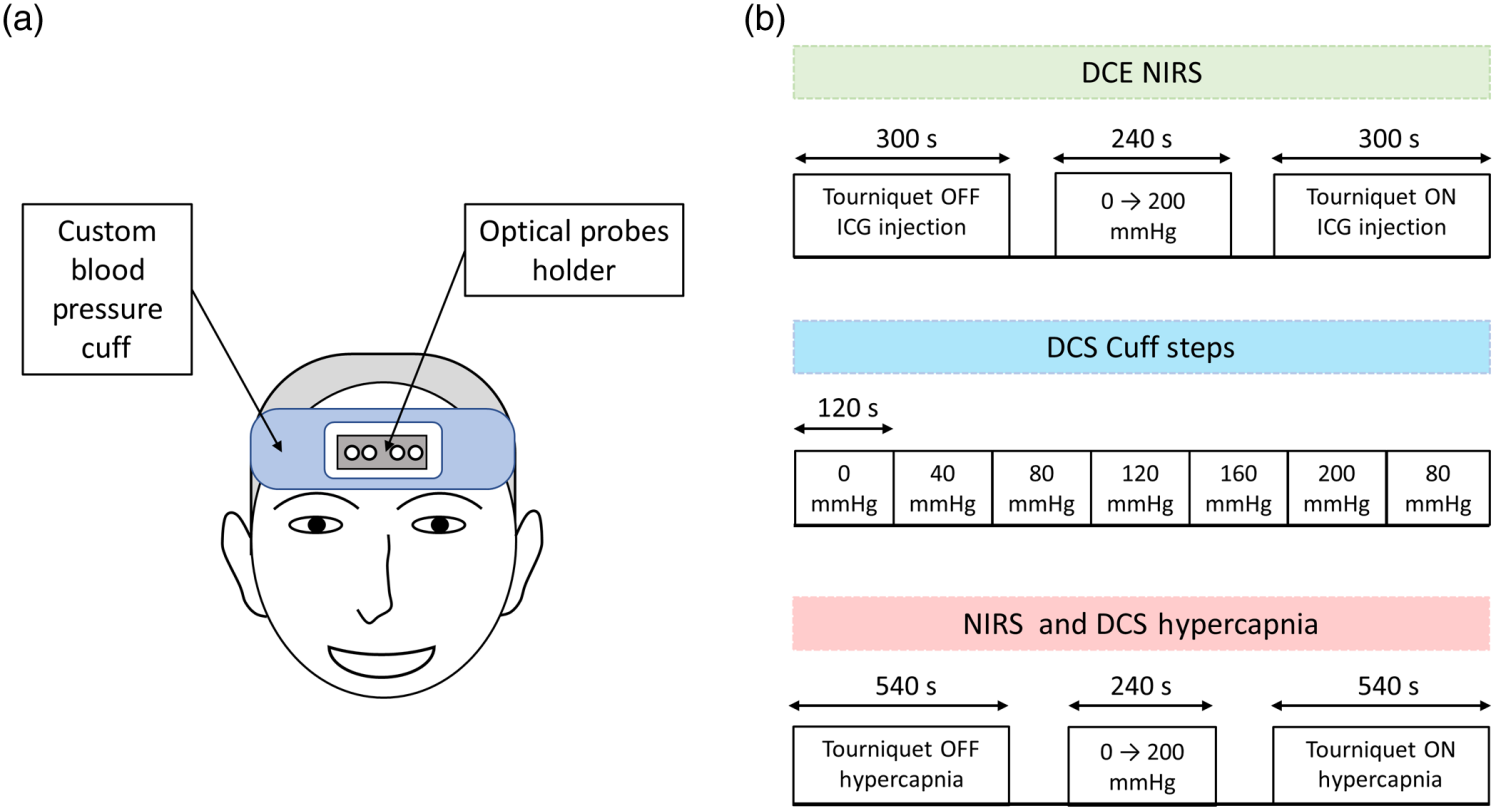
(a) Illustration of optical probes placement and (b) experimental paradigms used in the study.

A series of four separate experiments were conducted on each subject. All involved a 10-cm wide inflatable tourniquet wrapped around the head, just above the supraorbital ridge [Fig. 1(a)]. The tourniquet was designed to impede blood flow in the arteries supplying the scalp by temporally inflating two bladders positioned over the two temples. This would include the superficial temporal, supraorbital, and supratrochlear arteries supplying the forehead. The opening for the optical probes was 9×5  cm (length×width), which was designed to extend beyond the dimensions of the probe holder (7×3.5  cm) to ensure that inflating the tourniquet would not inadvertently change the ECL thickness by pressing down on the holder. The position of the opening in the tourniquet was adjusted on the forehead to locate the probes slightly off-center from the midline.

Both the positioning and space available for the probes were limited by the two bladders located over the temples. Confounding effects related to placing probes close to the sagittal sinus were considered; however, Liu and Chance et al.^49^ showed that NIRS measurements are primarily sensitive to oxygenation in the microvasculature as almost all light that interrogates larger vessels, such as the sagittal sinus, is absorbed. Placing the probes on the midline may have resulted in a greater loss of light, but the signals from both TR-NIRS and DCS were found to be adequate for this study. In three of the four experiments [two involving TR-NIRS and one involving DCS, Fig. 1(b)], data were acquired with and without manually inflating the tourniquet to 200 mmHg to impede SBF completely. The tourniquet was inflated rapidly to reach a new steady state. In addition, measurements after inflation were delayed by a few minutes to ensure that the condition and pressure were stable. No significant difference in the total hemoglobin for the tourniquet off (64.0±7.3  μM) and on (64.7±9.9  μM) was found. In the remaining experiment, DCS data were acquired across a series of tourniquet pressures ranging from 0 to 200 mmHg. Data were acquired at $r_{\mathrm{SD}}=1$, 3, and 4 cm for TR-NIRS and $r_{\mathrm{SD}}=1$ and 3 cm for DCS.

For the DCE-NIRS experiment, a catheter was inserted into an arm vein for injecting the optical contrast agent, ICG. The bolus tracking protocol involved a rapid intravenous injection of ICG (0.1  mg/kg), followed by acquiring a time series of DTOFs at a sampling frequency of 3 Hz and for 5 min to capture the kinetics of the dye. These data were acquired using an 803-nm laser, which is close to the maximum absorption of ICG. Two ICG injections were administered for every subject, one before inflating the tourniquet and the other after inflation. There was a delay of at least 15 min between injections to allow sufficient time for ICG to clear from the blood. The second ICG injection was administered 4 min after inflation to ensure stable pressure and to allow time for scalp hemodynamics to equilibrate.

**Fig. 2:**
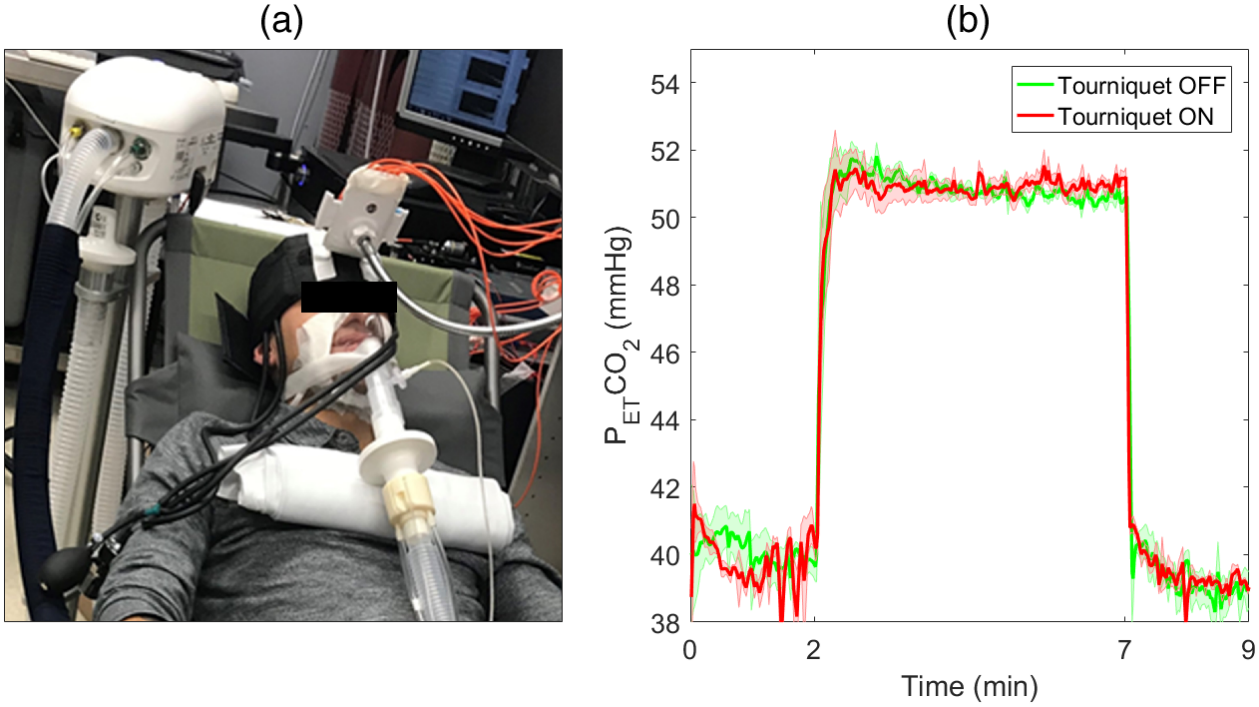
(a) Illustration of the experimental setup. The subject is wearing a sealed mask connected to the RespirAct™ system. An articulating arm stabilized the optical probes located on the forehead. (b) Time-varying changes in ${P_{\mathrm{ET}}\mathrm{CO}}_{2}$ recorded before and after the tourniquet was inflated.

The hypercapnia experiments involved recording either the oxygenation response or blood flow increase to a 5-min hypercapnic challenge, which was defined by an increase in the end-tidal partial pressure of carbon dioxide ($P_{\mathrm{ET}}{\mathrm{CO}}_{2}$) of 12 mmHg. A computerized gas control system (RespirAct™, Thornhill Research Inc., Toronto, Canada) was used to control $P_{\mathrm{ET}}{\mathrm{CO}}_{2}$, and subjects were required to wear a mask that was sealed by a transparent film dressing (Tegaderm™, 3M, St. Paul, United States) (Fig. 2). Similar to the DCE-NIRS experiments, the hypercapnia challenge was performed before and 4 min after tourniquet inflation. For the oxygenation experiment, DTOFs were recorded continuously for two lasers emitting at 760 and 830 nm. Sampling frequency was 3 Hz, and data were acquired for 9 min, which included a 2-min baseline period prior to hypercapnia and a 2-min recovery period after hypercapnia. For the blood flow experiment, DCD data were recorded at 3 Hz following the same protocol of 2-min baseline, 5-min hypercapnia, and 2-min recovery.

The last DCS experiment involved acquiring data during gradual increases in tourniquet pressure from 0 to 200 mmHg in steps of 40 mmHg. Each pressure value was maintained for 2 min, and the entire procedure lasted 14 min, during which DCS data were continuously acquired.

### 2.2 Instrumentation

The NIRS data were acquired using an in-house built, four-channel TR system.^28^^,^^50^ The system was equipped with picosecond pulsed lasers operated at three wavelengths λ=760, 803, and 830 nm, and at a pulse repetition rate of 80 MHz (PicoQuant, Berlin, Germany). For the 803-nm laser, the light pulses were coupled into a 2.5-m-long multimode fiber (ϕ=0.4  mm, NA=0.22, Fiberoptics Technology, Pomfret, Connecticut, United States). For the 760 and 830 nm lasers, light pulses from the two lasers’ heads were coupled into a 2.5-m long multimode bifurcated fiber (ϕ=0.4  mm, NA=0.39, Thorlabs, Newton, NJ, United States).^29^ Three detection fiber bundles (ϕ=3.6  mm, NA=0.22, Fiberoptics Technology, Pomfret, CT, United States) were held on a subject’s forehead using a 3-D-printed holder made of NinjaFlex© (NinjaTek, PA, United States). Diffusively reflected light from the surface of the head was delivered to hybrid photomultiplier tubes (PMA Hybrid 50, PicoQuant, Berlin, Germany) via the fiber bundles. A time-correlated single-photon counting unit (HydraHarp 400, PicoQuant, Berlin, Germany) was used to record the arrival times of the photons, and the corresponding DTOFs were built using LabView software.

DCS data were acquired with an in-house built system^7^^,^^51^ consisting of a long coherence length laser operated at 785 nm (CrystaLaser, Reno, NV, United States) and coupled to multimode fiber (ϕ=200  nm, NA=0.39, FT200UMT, Thorlabs, NJ, United States) to deliver the light to the head. Diffusely reflected light was collected by four single-mode fibers (SMF-28-J9, NA=0.14, core=8.2  μm, single-mode cutoff wavelength = 1260 nm, Thorlabs, NJ, United States), each coupled to a single-photon counting APDs (SPCM-AQR-15-FC, PerkinElmer Canada Inc., Quebec, Canada). In turn, each counting module generated TTL pulses that were sent to an edge-detecting photon counter on a PCIe6612 counter/timer data acquisition board (National Instrument, Austin, TX, United States).^52^ In-house developed software (LabVIEW, National Instrument, Austin, TX, United States) was used to record the total photon count and generate an intensity autocorrelation curve consisting of 40 delay times (τ) ranging from 1 to 40  μs.^7^ The resolution of data acquisition was fixed to 1  μs (f=60  MHz), and the number of bins was restricted to 40 (i.e., 40  μs) due to hardware memory limitations.

### 2.3 Data Analysis

Analysis of the TR-NIRS data for both the DCE-NIRS and hypercapnia experiments began by subtracting the background signal from each DTOF in a time series. The background signal was defined as the mean number of photons measured for the period before the initial rise of the DTOF. Next, the statistical moments [i.e., the total number of photons (N), mean time-of-flight (⟨t⟩), and variance (V)] were calculated for each DTOF using cut-off levels at 3% of the peak of the DTOF.^25^ Moment analysis was applied to the TR-NIRS data because it is a simple and robust method of obtaining depth sensitivity for measured DTOFs.^25^^,^^26^ The resulting times series were smoothed with a 4.5-s moving average with a zero-phase digital filter (filtfilt, MATLAB, MathWorks Inc., United States).

For the oxygenation hypercapnia experiments, baseline optical properties for each subject were estimated from the DTOFs collected during the period prior to the first hypercapnic challenge. The mean DTOF was fit with the solution to the diffusion equation for a semi-infinite homogeneous medium, using extrapolated boundary condition, after convolving with the measured IRF (fminsearch, MATLAB, Mathworks Inc., United States).^53^ The fitting range was set to 80% of the peak value of the DTOF on the leading edge and 20% on the falling edge. The fitting parameters were the absorption coefficient ($\mu_{a}$), the reduced scattering coefficient ($\mu_{s}^{\prime }$), and an amplitude factor. Next, the time series for each of the three moments was converted into the corresponding absorption changes $\Delta \mu_{a,i}(\lambda )$ by sensitivity analysis:^31^^,^^54^

$$\Delta \mu_{a,i}(\lambda )=\frac{\Delta m_{i}}{{\mathrm{SF}}_{i}},$$ (1)

where $\Delta m_{i}$ is the change in the i’th moment {$\Delta m_{0}=-\log[N(t)/N_{0}]$; $\Delta m_{1}=\langle t\rangle (t)-{\left\langle t\right\rangle }_{0}$; $\Delta m_{2}=V(t)-V_{0}$} and ${\mathrm{SF}}_{i}$ is the corresponding sensitivity factor derived from the diffusion approximation for a semi-infinitive homogenous model using each subject’s baseline optical properties. The absorption time courses determined at 760 and 830 nm were converted to changes in concentrations of oxyhemoglobin ($\Delta C_{\mathrm{HbO}}$) and deoxyhemoglobin ($\Delta C_{\mathrm{Hb}}$) by

$$\Delta \mu_{a,i}(\lambda )=\varepsilon_{\mathrm{HbO}}(\lambda )\Delta C_{\mathrm{HbO},i}+\varepsilon_{\mathrm{Hb}}(\lambda )\Delta C_{\mathrm{Hb},i},$$ (2)

where $\varepsilon_{\mathrm{HbO}}(\lambda )$ and $\varepsilon_{\mathrm{Hb}}(\lambda )$ are the molar extinction coefficients for oxy- and deoxyhemoglobin. This analysis results in time series for $\Delta C_{\mathrm{HbO}}$ and $\Delta C_{\mathrm{Hb}}$ derived from the change in the number of photons [$\Delta N=N(t)/N_{0}$], mean time-of-flight [$\Delta \langle t\rangle =\langle t\rangle (t)-{\left\langle t\right\rangle }_{0}$], and variance [$\Delta V=V(t)-V_{0}$].

Signal contamination from the ECL was estimated in the DCE-NIRS and hypercapnia experiments by comparing signal changes measured prior to and following tourniquet inflation. For DCE-NIRS, signal contrast was calculated as the maximum signal change following ICG injection relative to baseline. ECL contamination was defined as the percent difference between the two signal contrasts normalized by the contrast measured after inflation. For the hypercapnia experiments, signal contrast was defined by the average change in hemoglobin concentration for the last 2.5 min of the hypercapnic challenge. Contrast and ECL contamination were calculated separately for $\Delta C_{\mathrm{HbO}}$ and $\Delta C_{\mathrm{Hb}}$.

For DCS, the time series of temporal intensity fluctuations measured at each $r_{\mathrm{SD}}$ was used to compute the normalized intensity autocorrelation function ($g_{2}$).^55^ The $g_{2}$ curves (the entire range of τ values was used to include sensitivity to both scalp and brain) were analyzed in two ways. First, each $g_{2}$ curve was analyzed using the solution to the correlation diffusion equation for a semi-infinite homogenous medium. This analysis incorporated each individual’s optical properties derived from TR-NIRS and the coherence factor (β) determined from the average initial value of the baseline $g_{2}$ curves. The fitting procedure yielded a best-fit estimate of the BFI, which was based on modeling perfusion as a pseudo-Brownian motion.^6^ Similar to the TR-NIRS hypercapnia experiments, signal contrast was defined by the average change in BFI for the last 2.5 min of hypercapnia, and ECL contamination was determined by comparing signal contrast with and without inflating the tourniquet.

As a second analysis, $g_{2}$ curves from the two $r_{\mathrm{SD}}$ values were analyzed together using a three-layered DCS model that represents blood flow in the scalp, skull, and brain, separately.^16^ Estimating the ECL optical properties using TR data from a short source–detector separation is challenging; therefore, the optical properties for all three layers were taken from the literature for the fitting procedure^56^ ($\mu_{a,\text{skin}}=0.18\;{\mathrm{cm}}^{-1}$, $\mu_{a,\text{skull}}=0.16\;{\mathrm{cm}}^{-1}$, $\mu_{a,\text{brain}}=0.17\;{\mathrm{cm}}^{-1}$, $\mu_{s,\text{skin}}^{\prime }=\mu_{s,\text{skull}}^{\prime }=\mu_{s,\text{brain}}^{\prime }=12\;{\mathrm{cm}}^{-1}$), whereas average thicknesses of the scalp (5 mm) and skull (7 mm) layers were obtained from a previous study.^31^ It should be noted that the assumed optical properties were similar to reconstructed values presented in Sec. 3 and from previous studies.^31^

For each subject, β was calculated as the average maximum value of the $g_{2}$ curves measured at baseline. It should be noted that no change in the β value was observed following tourniquet inflation. The fitting parameters were blood flow indices for scalp and brain, assuming negligible flow in the middle (skull) layer. Since this fitting procedure was more computationally demanding than fitting the homogenous model, the $g_{2}$ curves measured at both $r_{\mathrm{SD}}$ values were averaged over 30-s intervals to improve the SNR.

The final step was to calculate the relative change in ${\mathrm{CMRO}}_{2}$ (${\mathrm{rCMRO}}_{2}$) during hypercapnia based on the standard mass balance equation relating ${\mathrm{CMRO}}_{2}$ to BFI and ${\mathrm{StO}}_{2}$:

$${\mathrm{rCMRO}}_{2}(t)=\frac{{\mathrm{CMRO}}_{2}(t)-{\mathrm{CMRO}}_{2}(0)}{{\mathrm{CMRO}}_{2}(0)}=\frac{\mathrm{BFI}(t)\cdot [100\%-{\mathrm{StO}}_{2}(t)]}{\mathrm{BFI}(0)\cdot [100\%-{\mathrm{StO}}_{2}(0)]}-1,$$ (3)

where Eq. (3) implicitly assumes that ${\mathrm{StO}}_{2}$ is comprised of constant arterial and venous fractions^57^^,^^58^ and arterial oxygen saturation is 100%. Baseline values are denoted by t=0, and ${\mathrm{StO}}_{2}$ was determined using:

$${\mathrm{SO}}_{2}(t)=\frac{C_{\mathrm{HbO}}(0)+\Delta C_{\mathrm{HbO}}(t)}{\left[C_{\mathrm{HbO}}(0)+\Delta C_{\mathrm{HbO}}(t)]+[C_{\mathrm{Hb}}(0)+\Delta C_{\mathrm{Hb}}(t)\right]},$$ (4)

where $C_{\mathrm{HbO}}(0)$ and $C_{\mathrm{Hb}}(0)$ were determined using each subject’s baseline optical properties and assuming a water content of 75%. $\Delta C_{\mathrm{HbO}}$ and $\Delta C_{\mathrm{Hb}}$ were derived from the ΔV time series measured at $r_{\mathrm{SD}}=4\;\mathrm{cm}$ since this combination of distance and statistical moment provides the greatest depth sensitivity. BFI(t) was determined either from the largest source–detector separation ($r_{\mathrm{SD}}=3\;\mathrm{cm}$) or from the CBF values obtained from the multilayer model.

### 2.4 Statistical Analysis

All data are presented as mean ± standard error of the mean unless otherwise noted. Statistical significance was defined as p<0.05. A paired t-test was used to assess differences between optical properties before and after inflating the head tourniquet. An n-way analysis of variance (ANOVA) was used to investigate differences in ECL signal contamination for TR-NIRS data acquired at the three $r_{\mathrm{SD}}$ values and derived from the three statistical moments of the DTOFs. This analysis was conducted individually for ICG, $\Delta C_{\mathrm{HbO}}$, and $\Delta C_{\mathrm{Hb}}$ data sets. A one-way ANOVA was used to evaluate the changes in BFI in response to incremental increases in tourniquet pressure. This analysis was conducted for data recorded at the two $r_{\mathrm{SD}}$ separately as well as for the CBF values derived from the multilayer model. Finally, paired t-tests were used to determine if there were significant changes in BFI and ${\mathrm{rCMRO}}_{2}$ in response to the hypercapnic challenge. These tests were conducted by compare average values for the 2-min baseline period and the last 2.5 min of hypercapnia.

## 3 Results

Average optical properties across the five subjects before tourniquet inflation were $\mu_{a}=(0.14\pm 0.02)\;{\mathrm{cm}}^{-1}$ and $\mu_{s}^{\prime }=(10.3\pm 2.5)\;{\mathrm{cm}}^{-1}$ at 760 nm and $\mu_{a}=(0.15\pm 0.03)\;{\mathrm{cm}}^{-1}$ and $\mu_{s}^{\prime }=(9.5\pm 2.5)\;{\mathrm{cm}}^{-1}$ at 830 nm. After tourniquet inflation, $\mu_{a}=(0.15\pm 0.03)\;{\mathrm{cm}}^{-1}$ and $\mu_{s}^{\prime }=(10.8\pm 1.45)\;{\mathrm{cm}}^{-1}$ at 760 nm and $\mu_{a}=(0.15\pm 0.05)\;{\mathrm{cm}}^{-1}$ and $\mu_{s}^{\prime }=(9.8\pm 2.5)\;{\mathrm{cm}}^{-1}$ at 830 nm. There was no significant difference in either $\mu_{a}$ or $\mu_{s}^{\prime }$ due to inflating the tourniquet.

**Fig. 3:**
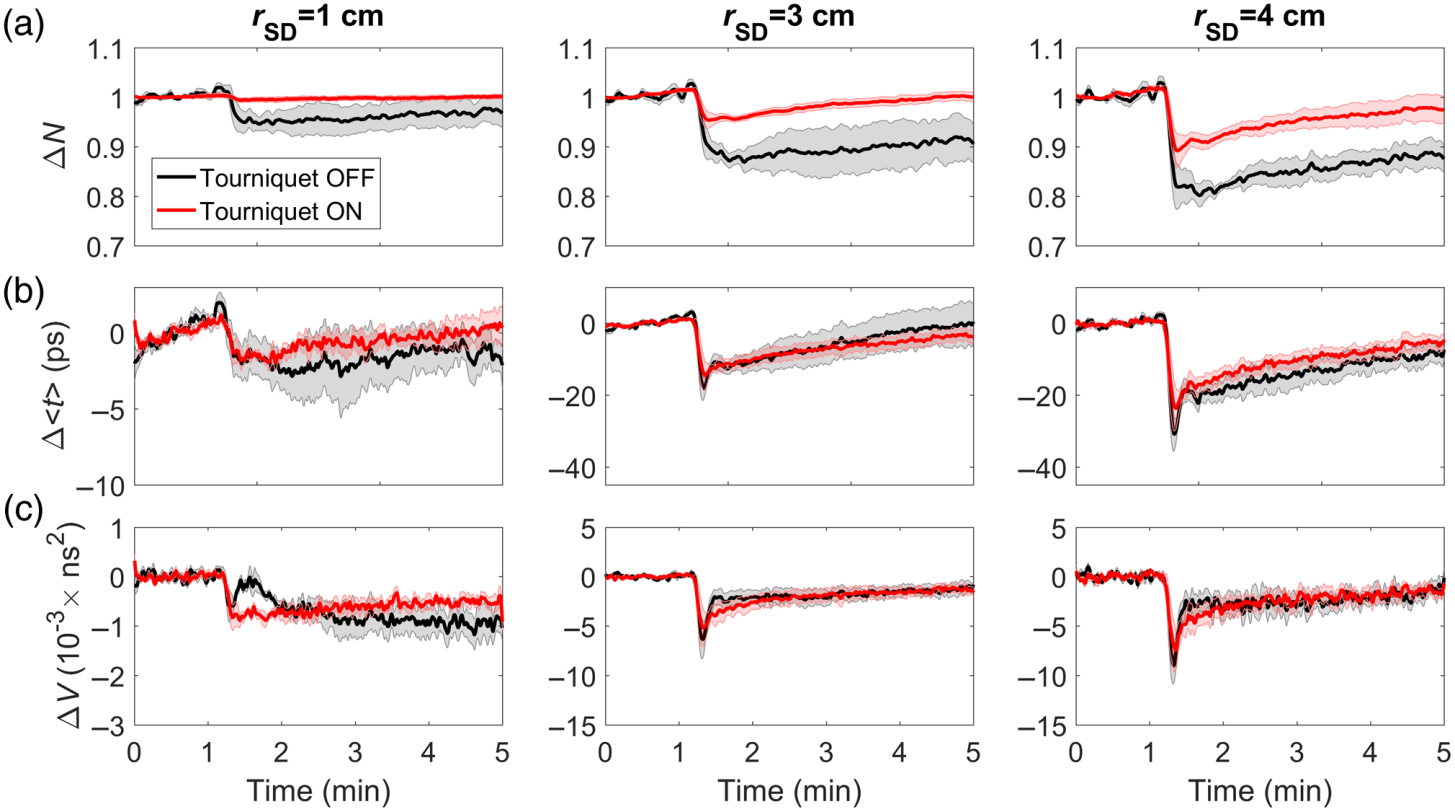
Change in the (a) number of photons, (b) mean time-of-flight, and (c) variance plotted as a function of time following an intravenous bolus injection of ICG. Time courses are shown before (black) and after (red) inflating the tourniquet and for three source–detector distances. Shading surrounding each line represents the standard error of the mean.

Average time courses of ΔN, Δ⟨t⟩, and ΔV following the intravenous bolus injection of ICG are presented in Fig. 3. Note that the ΔN and Δ⟨t⟩ time courses were presented previously,^31^ but the ΔV analysis was not. There are observable differences between ICG time courses obtained before and after tourniquet inflation, especially for ΔN. The lack of a change in ΔN at $r_{\mathrm{SD}}=1\;\mathrm{cm}$ after the tourniquet was inflated demonstrates its effectiveness at blocking SBF. In contrast, the passage of ICG through the scalp had minimal effect on the ΔV time course for $r_{\mathrm{SD}}=1\;\mathrm{cm}$ prior to inflation, demonstrating the insensitivity of the variance to the superficial tissue. It should be noted that the difference in dynamics of the ΔV curves measured with and without the tourniquet ($r_{\mathrm{SD}}=3$ and 4 cm) is due to the difference in amplitude of the first pass. This effect diminishes by normalizing the two curves, and any residual differences are likely due to variations in the manual injections.

Estimates of signal contamination from the ECL for the three source–detector separations and statistical moments are presented in Table 1. Reflecting the trends observable in Fig. 3, signal contamination decreased with increasing source–detector separation and order of the statistical moment. The ECL signal contamination was significantly different between signals measured at $r_{\mathrm{SD}}=1$ and ≥3  cm for all moments. In addition, a statistically significant decrease in ECL contamination was observed for Δ⟨t⟩ and ΔV compared with ΔN for all $r_{\mathrm{SD}}$. There were no significant differences between ECL contamination for Δ⟨t⟩ and ΔV.

**Table 1:** Average estimates of ECL signal contamination from the DCE-NIRS experiment.

	ECL signal contamination (%)		
	1 cm	3 cm	4 cm
ΔN	81±8	52±9	39±8
Δ⟨t⟩	40±6	12±3	8±6
ΔV	−30±11	8±5	8±4

**Fig. 4:**
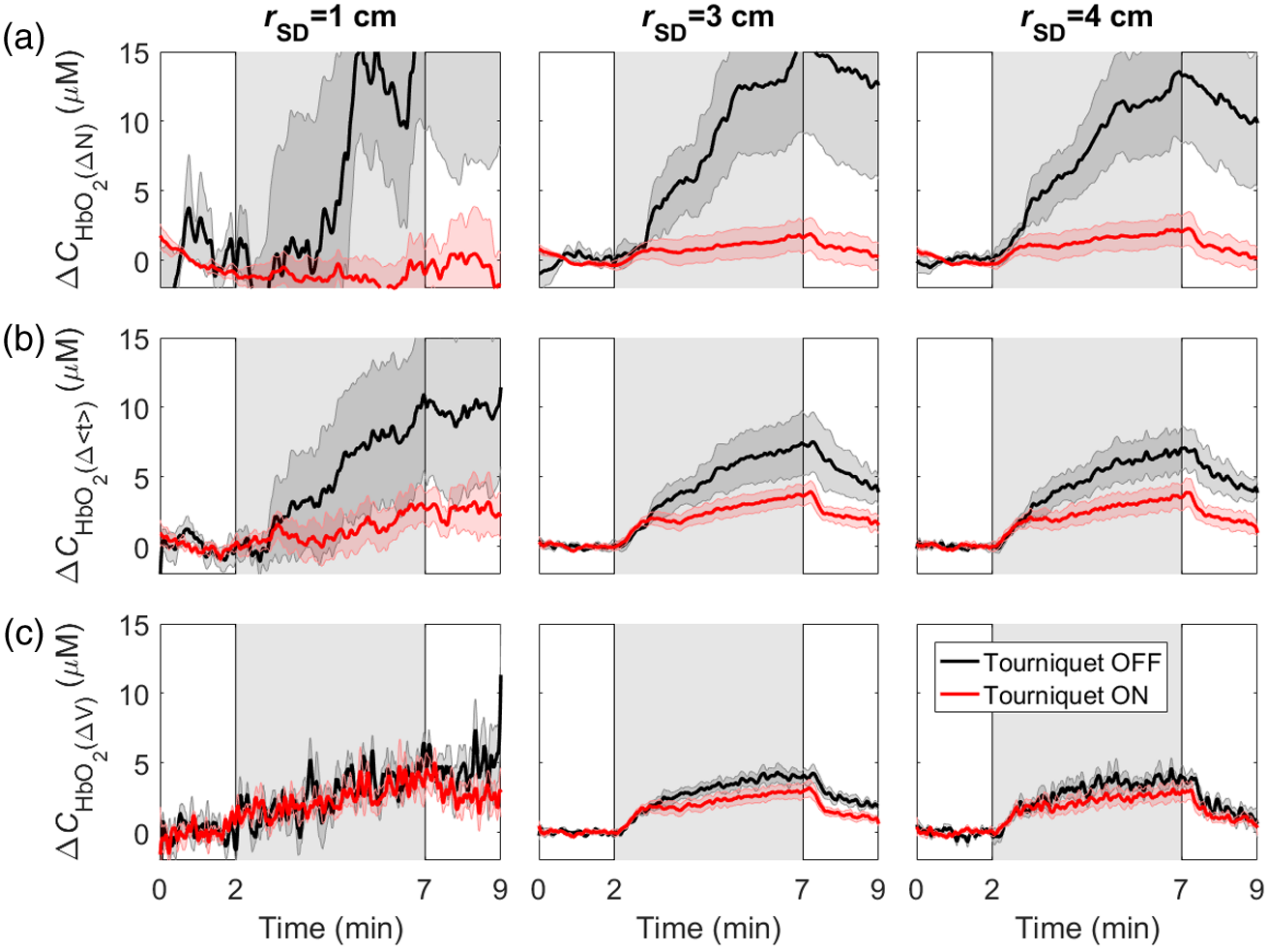
Average time courses of the change in oxyhemoglobin concentration ($\Delta C_{\mathrm{HbO}}$) in response to hypercapnia, which is indicated by the gray region between 2 and 7 min. Time courses are shown for data acquired before and after tourniquet inflation and determined from the (a) number of photons, (b) mean time-of-flight, and (c) variance. Shading surrounding each line represents the standard error of the mean.

**Fig. 5:**
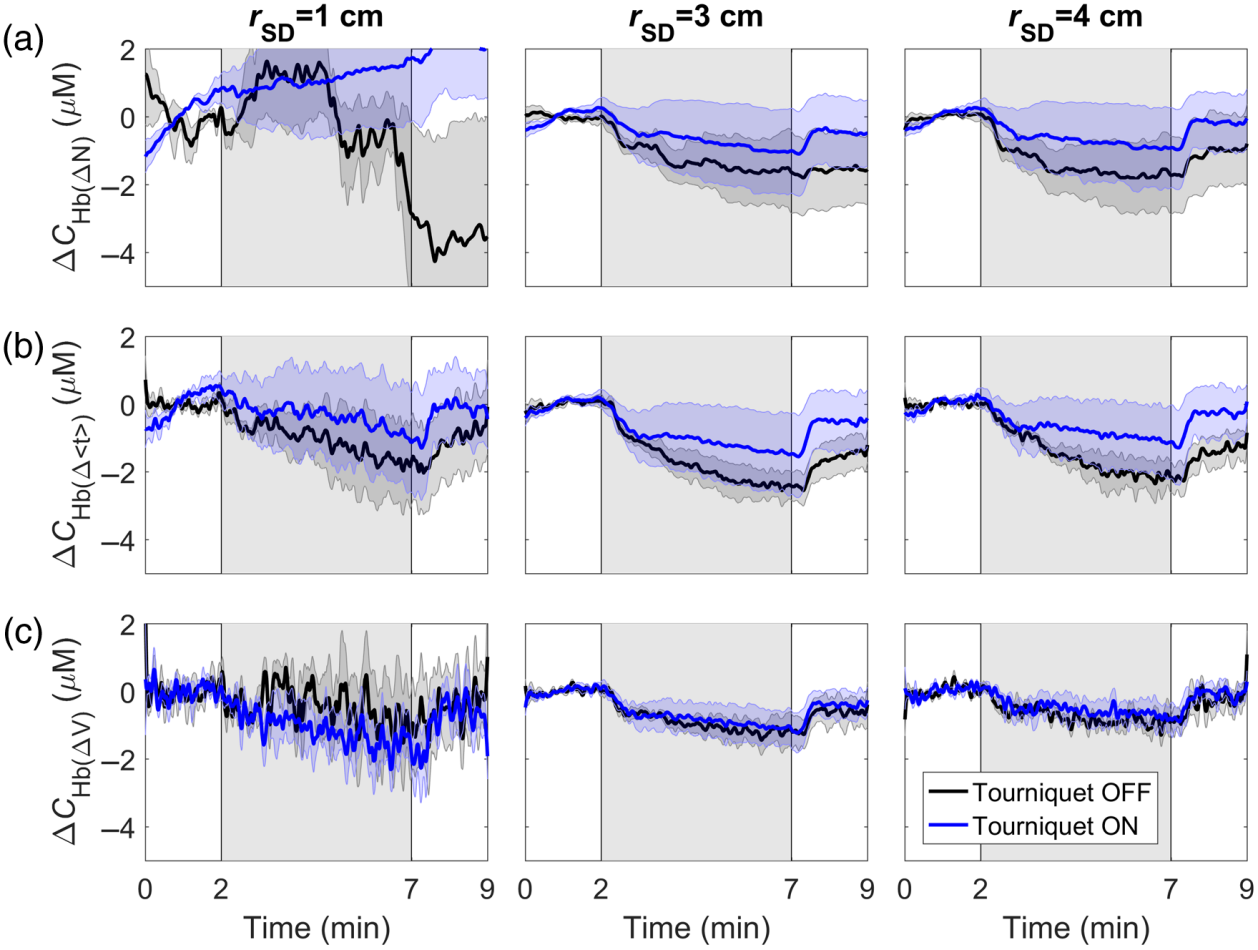
Average time courses of the change in deoxyhemoglobin concentration ($\Delta C_{\mathrm{Hb}}$) in response to hypercapnia (gray region). Time courses are shown for data acquired before and after tourniquet inflation and determined from the (a) number of photons, (b) mean time-of-flight, and (c) variance. Shading surrounding each line represents the standard error of the mean.

The time-varying changes in $\Delta C_{\mathrm{HbO}}$ and $\Delta C_{\mathrm{Hb}}$ caused by hypercapnia are presented in Fig. 4 and 5, respectively. The results are presented with and without inflating the tourniquet for the three source–detector distances and three statistical moments. Similar to the DCE-NIRS results, differences between responses before and after tourniquet inflation were reduced as the order of the statistical moments increased. Comparing the two figures illustrates that $\Delta C_{\mathrm{Hb}}$ was less sensitive to scalp changes than $\Delta C_{\mathrm{HbO}}$. This result is reflected in the ECL signal contamination estimates for the two hemoglobin signals provided in Table 2. A significant decrease in ECL contamination was observed for $\Delta C_{\mathrm{HbO}}$ derived from ΔV compared with $\Delta C_{\mathrm{HbO}}$ derived from ΔN for all $r_{\mathrm{SD}}$. No statistically significant difference between the ECL contamination in $\Delta C_{\mathrm{Hb}}$ signals was observed.

**Table 2:** Average estimates of signal contamination from the ECL during hypercapnia.

	ECL signal contamination (%)					
	$\Delta C_{\mathrm{HbO}}$			$\Delta C_{\mathrm{Hb}}$		
	1 cm	3 cm	4 cm	1 cm	3 cm	4 cm
ΔN	90±8	89±15	83±11	—	61±29	50±31
Δ⟨t⟩	77±29	51±12	49±7	61±22	43±25	42±21
ΔV	20±15	30±8	28±11	48±26	15±27	8±38

Comparing all time courses in Figs. 4 and 5, the higher noise level for the number of photons measured at $r_{\mathrm{SD}}=1\;\mathrm{cm}$ was attributed to large scalp signal variations between subjects. Since the higher moments (especially the variance) are not as sensitive to the scalp, this intersubject variability was not as great. The sensitivity to the scalp at 1 cm is also evident by comparing the time courses with and without inflating the tourniquet.

**Fig. 6:**
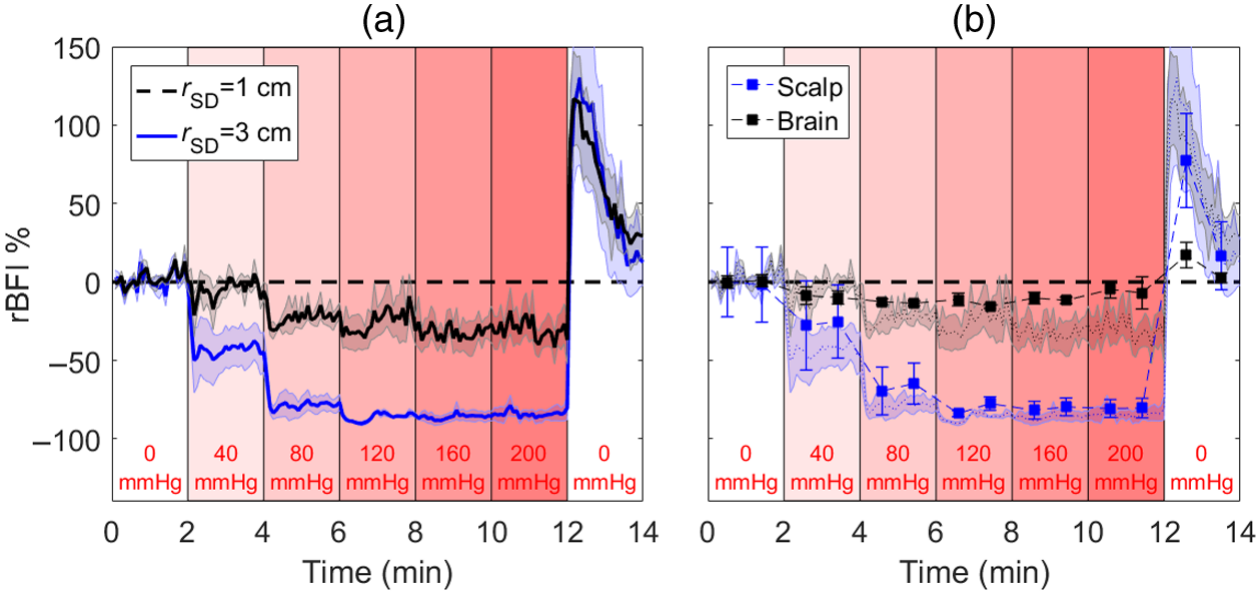
(a) Changes in the blood flow index (rBFI) in response to step increases in tourniquet pressure recorded at $r_{\mathrm{SD}}=1$ and 3 cm. Shading surrounding each line represents the standard error of the mean. (b) BFI values for brain and scalp were derived using the multilayer model. Error bars represent the standard error of the mean. For reference, the shading represents the individual time courses [from (a)] recorded at $r_{\mathrm{SD}}=1$ and 3 cm.

Relative changes in the blood flow index (rBFI) in response to incremental increases in tourniquet pressure are presented in Fig. 6(a). Time courses of rBFI are presented for the two source–detector distances separately. Larger flow reductions were observed, even at lower applied pressures. The effects of the tourniquet were larger for the data recorded at $r_{\mathrm{SD}}=1\;\mathrm{cm}$ with a maximum change in BFI of (−85±2)% for pressures higher than 120 mmHg. The corresponding change recorded at $r_{\mathrm{SD}}=3\;\mathrm{cm}$ was (−31±5)%. Statistically significant decreases in BFI were found for both $r_{\mathrm{SD}}$ at all tourniquet pressures. Figure 6(b) presents scalp and CBF estimates obtained by analyzing the two time-courses simultaneously by the multilayer DCS model. A relatively small change in CBF was found (−9±3)% at the highest two pressures, whereas the corresponding change in SBF was (−81±1)%. Significant decreases were found for SBF for pressures >40  mmHg and for CBF for pressures of 80 and 120 mmHg only.

**Fig. 7:**
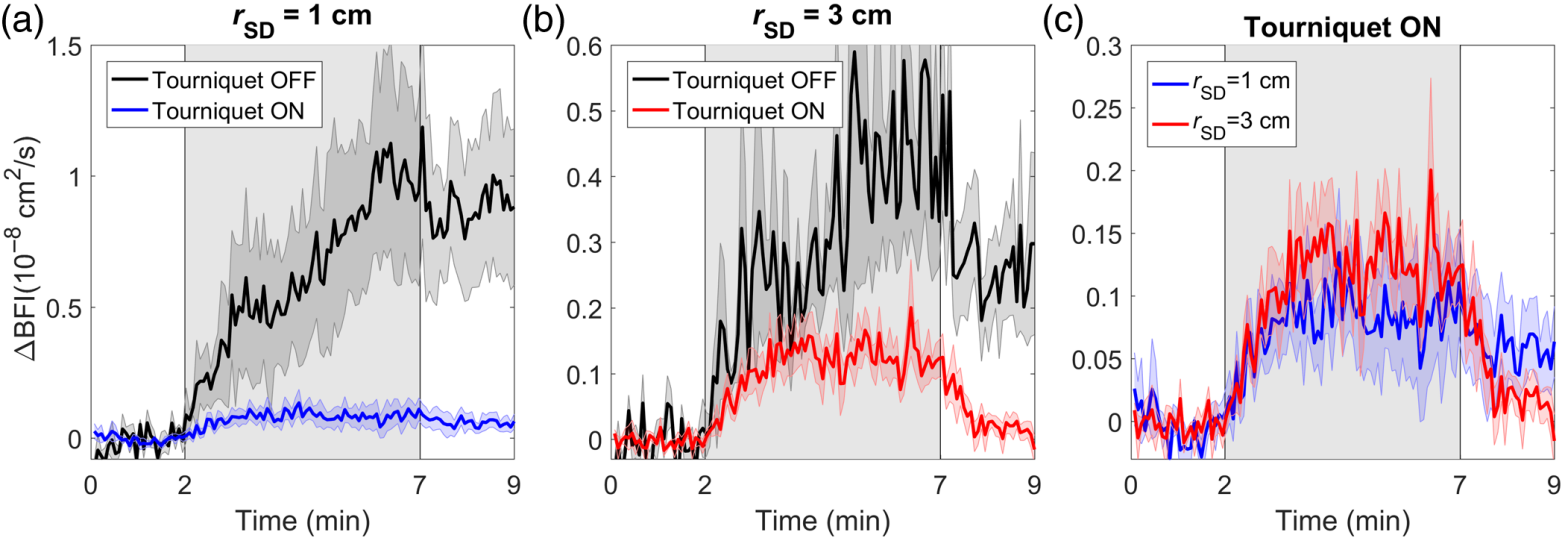
Changes in blood flow index (ΔBFI) plotted as a function of time during the hypercapnic challenge with and without tourniquet inflation. Time courses are presented for (a) $r_{\mathrm{SD}}=1\;\mathrm{cm}$ and (b) $r_{\mathrm{SD}}=3\;\mathrm{cm}$. (c) For illustration purposes, the ΔBFI time courses for the two distances when the tourniquet was inflated are repeated. The shadowing represents the standard error of the mean.

Time-varying changes in BFI (ΔBFI) in response to the hypercapnic challenge are presented in Fig. 7. When the tourniquet was not inflated, ΔBFI measured at $r_{\mathrm{SD}}=1\;\mathrm{cm}$ showed a persistent elevation after hypercapnia, similar to $\Delta C_{\mathrm{HbO}}$ derived from ΔN. These results indicate that the DCS signal measured at the short $r_{\mathrm{SD}}$ predominately reflected changes in scalp hemodynamics. After inflating the tourniquet, the ΔBFI time courses measured at both distances were similar in shape, demonstrating that, even at $r_{\mathrm{SD}}=1\;\mathrm{cm}$, there was some sensitivity to CBF. However, this brain contribution is small and overshadowed by changes in SBF when the tourniquet was not inflated. ECL contamination for ΔBFI was (75±18)% at $r_{\mathrm{SD}}=1\;\mathrm{cm}$ and (48±18)% at 3 cm. There was no statistically significant difference between these values.

**Fig. 8:**
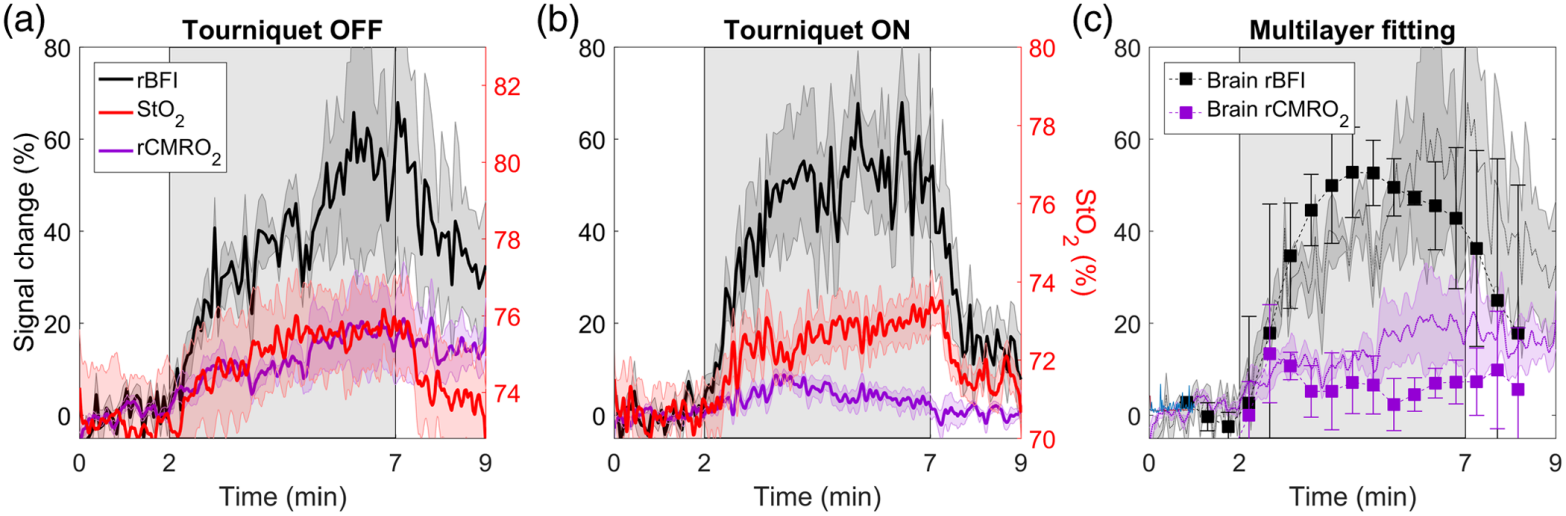
Tissue saturation (${\mathrm{StO}}_{2}$), rBFI, and ${\mathrm{rCMRO}}_{2}$ during the hypercapnic challenge (a) without and (b) with the tourniquet inflated. All data were acquired at $r_{\mathrm{SD}}=3\;\mathrm{cm}$, and ${\mathrm{StO}}_{2}$ was derived from the variance. Shadowing represents the standard error of the mean. (c) Final graph presents relative CBF changes obtained from the multilayer model and the corresponding ${\mathrm{rCMRO}}_{2}$. Error bars represent the standard error of the mean, and the shading represents rBFI and ${\mathrm{rCMRO}}_{2}$ time series with the tourniquet not inflated.

Average time courses of ${\mathrm{StO}}_{2}$ and relative changes in BFI (rBFI) and ${\mathrm{CMRO}}_{2}$ (${\mathrm{rCMRO}}_{2}$) in response to hypercapnia are presented in Fig. 8. ${\mathrm{StO}}_{2}$ was derived from the variance signal, and both ${\mathrm{StO}}_{2}$ and BFI (rBFI) time courses were acquired at $r_{\mathrm{SD}}=3\;\mathrm{cm}$. Without inflating the tourniquet, there was a significant increase of 20% in ${\mathrm{CMRO}}_{2}$ during the second half of the challenge. After inflating the tourniquet, hypercapnic ${\mathrm{rCMRO}}_{2}$ remained below 10% and did not reach statistical significance. Similarly, ${\mathrm{rCMRO}}_{2}$ did not reach significance when using the multilayer approach to calculate the change in CBF from the tourniquet-off data. Note that a small reduction in baseline ${\mathrm{StO}}_{2}$ was observed after inflating the tourniquet. This was likely a consequence of extracting the baseline optical properties using the homogenous solution to the diffusion approximation.

## 4 Discussion

This study focused on evaluating methods of reducing ECL signal contamination on NIRS and DCS measurements of ${\mathrm{StO}}_{2}$ and CBF, which were combined to determine ${\mathrm{CMRO}}_{2}$. The motivation was to improve the confidence in these noninvasive optical techniques for applications in critical-care settings, given the clinical interest in using flow and metabolic markers to help reduce the incidence of secondary brain injury.^59^ The concept of combining NIRS and DCS to monitor CBF and ${\mathrm{CMRO}}_{2}$ is well known;^58^ however, there have been few studies involving adult patients, in contrast to the number of studies involving neonatal patient populations.^4^^,^^5^^,^^60^ This difference reflects the challenges of adapting these technologies to neuromonitoring of adults due primarily to ECL signal contamination. In this study, ECL impact was evaluated by directly impeding blood flow to the scalp using a tourniquet. The effectiveness of this approach was demonstrated by the large change in ΔN measured at $r_{\mathrm{SD}}=1\;\mathrm{cm}$ in response to injecting ICG (Fig. 3), considering this measure had the greatest sensitivity to the scalp. On average, inflating the tourniquet reduced the ΔN response by (81±8)%. The fact that the ΔN response recorded at 3 and 4 cm did not mirror the corresponding responses extracted from the higher moments likely reflects some residual effects of SBF.

The advantage of TR detection for improving the depth sensitivity of NIRS is evident in both the DCE and oxygenation data sets. Differences between signals measured with and without inflating the tourniquet diminished as the source–detector distance and order of the moment increased, which indicates less signal contribution from the ECL. Across the three separate measures (DCE, $\Delta C_{\mathrm{HbO}}$, and $\Delta C_{\mathrm{Hb}}$), ECL contamination decreased by 3 to 6 times for ΔV compared with ΔN at $r_{\mathrm{SD}}=3$ and 4 cm (Tables 1 and 2). This difference in ECL sensitivity is also evident by comparing the shapes of the DCE and hypercapnic responses across the three separate moments. The primary feature of the mean DCE response determined from ΔV is its sharp profile during the first pass of ICG, which reflects the rapid movement of the dye through the cerebral microvasculature due to high blood flow.^31^ This feature was not as distinct in the corresponding ΔN response, and the clearance of the dye was much slower, reflecting the lower blood flow in scalp.

The hemodynamic responses to hypercapnia also exhibited noticeable differences between the statistical moments at the different source–detector distances (Figs. 4 and 5). At $r_{\mathrm{SD}}=1\;\mathrm{cm}$, the tourniquet abolished the $\Delta C_{\mathrm{HbO}}$ hypercapnic response derived from ΔN, whereas observable responses for Δ⟨t⟩ and ΔV were still evident. These patterns indicate that the higher moments were sensitive to the brain even at this short distance. More importantly, the effects of the tourniquet on $\Delta C_{\mathrm{HbO}}$ and $\Delta C_{\mathrm{Hb}}$ at $r_{\mathrm{SD}}=3$ and 4 cm greatly diminished for ΔV compared with the lower moments. The similarity between responses measured with and without the tourniquet inflated demonstrate the insensitivity of ΔV to the scalp. In contrast, the corresponding ΔN time courses measured before tourniquet inflation exhibited a persistent increase in $\Delta C_{\mathrm{HbO}}$ and decrease in $\Delta C_{\mathrm{Hb}}$ after the hypercapnic challenge, despite $P_{\mathrm{ET}}{\mathrm{CO}}_{2}$ rapidly returning to normocapnia (Fig. 1). The changes in scalp hemodynamics likely resulted from hypercapnia-induced increases in heart rate and blood pressure.^61^ These indicate that a latent vasodilatory response to hypercapnia in the scalp considering functional magnetic resonance imaging studies have shown rapid cerebrovascular reactivity in the healthy brain.^62^^–^^64^ Across all three moments, the ECL contribution was consistently smaller for $\Delta C_{\mathrm{Hb}}$ compared with $\Delta C_{\mathrm{HbO}}$, indicating that the former was less sensitive to scalp hemodynamics. This finding is in agreement with the results of our previous study that applied moment analysis to functional NIRS data.^61^ The greater sensitivity of deoxyhemoglobin to cerebral hemodynamics also agrees with a previous study by Kirilina et al.^65^

The temporal fluctuations in the time courses presented in Figs. 4 and 5 illustrate that the SNR decreased as the order of the statistical moment increased, which is expected, given the fewer number of late-arriving photons detected. As an example, the SNR for $C_{\mathrm{HbO}}$ measured at $r_{\mathrm{SD}}=4\;\mathrm{cm}$ was 84±11 for ΔN versus 34±5 for ΔV. Gated detectors could potentially improve the SNR of late photons by optimizing the laser power.^66^ However, these figures highlight that the reproducibility of the hypercapnic response across subjects improved as the order of the moment increased, despite the lower SNR. This is demonstrated by the SEM shown by the shaded region surrounding each time course. For $\Delta C_{\mathrm{HbO}}$, the between-subject SEM was ∼6 times smaller for ΔV compared with ΔN (0.64  μM versus 3.77  μM, respectively). This indicates that the greatest source of variability across subjects was variations in scalp contributions. This is confirmed by the tourniquet results, particularly for $\Delta C_{\mathrm{HbO}}$, which exhibited noticeably less intersubject variability than their tourniquet-off counterparts.

Although TR detection has been proposed for DCS,^33^^,^^67^ depth sensitivity in this study was investigated by collecting CW DCS data at two source-detector distances, and the BFI time courses acquired at $r_{\mathrm{SD}}=1$ and 3 cm did exhibit different depth sensitivities. This is evident in the data acquired with different tourniquet pressures [Fig. 6(a)] and by comparing BFI hypercapnic responses measured with and without the tourniquet (Fig. 7). Regarding the former, the reductions in BFI with increasing pressure were considerably larger at $r_{\mathrm{SD}}=1\;\mathrm{cm}$ compared with 3 cm [maximum change of (−85±2)% and (−31±5)%, respectively]. However, the significant changes measured at $r_{\mathrm{SD}}=3\;\mathrm{cm}$ indicate that even at this larger $r_{\mathrm{SD}}$ the DCS signal was susceptible to the influence of the ECL and should not be treated as a marker of CBF alone. This is also evident by comparing BFI hypercapnic responses shown in Fig. 7. Similar to the lower-moment oxygenation data, the tourniquet substantially reduced the magnitude of the BFI increase during hypercapnia and eliminated the residue signal after $P_{\mathrm{ET}}{\mathrm{CO}}_{2}$ had returned to normocapnia.

To separate SBF from CBF, we applied a multilayer model that was previously validated in a porcine model by comparison with CBF measurements acquired with perfusion computed tomography.^16^ The model largely removed from the CBF estimates the effects of transient scalp ischemia caused by the tourniquet, including the larger hyperemic response when the tourniquet pressure was fully released [Fig. 6(b)]. These results are in good agreement with Baker et al., who conducted a similar study that involved applying a two-layer Beer–Lambert law to MD DCS data.^37^ In this study, the multilayer model was further evaluated by applying it to hypercapnic data acquired with the tourniquet off (Fig. 8). The ratio of the deep-to-superficial baseline BFI values (calculated for the period before hypercapnia) was 3.9. This confirms that the model was able to reconstruct correct relative sensitivities in the different layers of the model. Despite the lower temporal resolution of the CBF time course due to temporal averaging, the predicted hypercapnic CBF response was similar in magnitude and shape to the BFI response recorded with the tourniquet inflated. This included the lack of a posthypercapnia residual seen in the individual BFI time courses (Fig. 7). Further improvement to the two-layer model could be achieved by incorporating individual measurements of the scalp/skull thickness, although this would require structural imaging.

ECL contamination was also evident in the ${\mathrm{rCMRO}}_{2}$ time courses presented in Fig. 8. Without inflating the tourniquet, the calculated ${\mathrm{rCMRO}}_{2}$ time course steadily increased during hypercapnia, reaching a maximum of roughly 20%. After inflating the tourniquet, temporal changes in ${\mathrm{rCMRO}}_{2}$ were smaller (<10%) and not statistically significant, as expected since hypercapnia at these levels does not affect metabolism.^68^ The similarity between the ${\mathrm{StO}}_{2}$ time courses measured with and without the tourniquet indicates that contamination from scalp was small for ${\mathrm{StO}}_{2}$ estimates derived from ΔV. The cause of the error in the first case was more likely a result of a slow SBF hypercapnic response, leading to an imbalance in Eq. (3) between changes in blood flow and cerebral oxygen extraction. As a final step, ${\mathrm{rCMRO}}_{2}$ was calculated using the CBF time course derived by applying the multilayer model to the tourniquet-off DCS data. In this case, ${\mathrm{rCMRO}}_{2}$ did not reach significance during hypercapnia, in agreement with the tourniquet-on results.

The data presented in Figs. 7 and 8 highlight the fundamentally poorer SNR of DCS compared with NIRS, which necessitated substantial signal averaging to generate stable BFI measurements from the multilayer model. In this study, the autocorrelation curves were collected over a relatively narrow range of delay times (1 to 40  μs). Using a wider range would help improve the delineation between brain and scalp contributions, thereby reducing the SNR requirements in the fitting procedure. Additionally, the sensitivity of DCS to CBF at $r_{\mathrm{SD}}=1\;\mathrm{cm}$ suggests that the longer source–detector distance could be slightly shorter than 3 cm to enhance SNR while still providing adequate depth sensitivity. Optimization of these timing and acquisition parameters could improve the stability of CBF estimates extracted from MD DCS data. As a final point, recently proposed subtraction-based methods for moment analysis have the potential to further enhance the depth sensitivity of oxygenation measurements.^69^^–^^72^

Although the brain is extremely sensitive to hypercapnia, vasodilation can occur in other tissues, including muscle.^73^^,^^74^ Changes in heart rate and blood pressure caused by the hypercapnic challenge could also lead to changes in blood flow and oxygenation in peripheral tissues. In a recent publication,^61^ significant increases in both parameters were caused by a $P_{\mathrm{ET}}{\mathrm{CO}}_{2}$ increase of 15 mmHg. Consider the inherent sensitivity of these optical techniques to superficial tissue, hemodynamic responses in scalp tissue would not have to be as large as cerebrovascular responses to cause significant signal changes. These results are in contrast to a previous study that reported no change in SBF, as measured by laser Doppler.^75^ This discrepancy is likely explained by the limited depth sensitivity of laser Doppler (depth<1  mm), which is primarily sensitive to skin blood flow. NIRS recordings at $r_{\mathrm{SD}}=1\;\mathrm{cm}$, on the other hand, would be sensitive to the entire scalp layer, which is highly vascularized. This difference between laser Doppler and NIRS signals was previously reported in a study of functional activation.^65^

## 5 Conclusions

In summary, TR-NIRS data collected with and without temporary scalp ischemia demonstrated how moment analysis could substantially reduce the effects of ECL contamination on cerebral oxygenation measurements. Similarly, it was shown that SBF and CBF responses to hypercapnia could be separated using a multilayer model to analyze DCS data acquired at two source–detector distances. These depth-enhanced techniques were combined to measure ${\mathrm{CMRO}}_{2}$ during hypercapnia and, as expected, no significant metabolic changes were measured despite large increases in CBF and ${\mathrm{StO}}_{2}$. These results highlight the potential of these noninvasive optical techniques for neuromonitoring applications.
